# Proton Therapy Reduces the Effective Dose to Immune Cells in Mediastinal Hodgkin Lymphoma Patients

**DOI:** 10.1016/j.ijpt.2024.100110

**Published:** 2024-06-20

**Authors:** Pierre Loap, Ludovic De Marzi, Justine Decroocq, Rudy Birsen, Natacha Johnson, Benedicte Deau Fischer, Didier Bouscary, Youlia Kirova

**Affiliations:** 1 Department of Radiation Oncology, Institut Curie, Paris, France; 2 Laboratoire d′Imagerie Translationnelle en Oncologie (LITO), Institut Curie, Université PSL, Université Paris-Saclay, Inserm U1288, Orsay, France; 3 Department of Hematology, Hopital Cochin, Paris, France

**Keywords:** Immune system, Hodgkin lymphoma, VMAT, Intensity-modulated proton therapy

## Abstract

**Purpose:**

Effective dose to circulating immune cells (EDIC) is associated with survival in lung and esophageal cancer patients. This study aimed to evaluate the benefit of intensity-modulated proton therapy (IMPT) for EDIC reduction compared with volumetric modulated arc therapy (VMAT) in mediastinal Hodgkin lymphoma (mHL) patients.

**Materials and Methods:**

Ten consecutive mHL patients treated with involved-site IMPT after frontline chemotherapy were included. The mean dose to the heart, lung, and liver and the integral dose to the body were obtained, and we calculated EDIC based on these variables. The effective dose to circulating immune cells was compared between IMPT and VMAT schedules.

**Results:**

The median EDIC was reduced from 1.93 Gy (range: 1.31-3.87) with VMAT to 1.08 Gy (0.53-2.09) with IMPT (*P* < .01). Integral dose reduction was the main driver of EDIC reduction with IMPT, followed by lung sparing.

**Conclusion:**

Intensity-modulated proton therapy significantly reduced EDIC in mHL patients undergoing consolidation involved-site radiation therapy. Integral dose reduction combined with improved lung sparing was the main driver of EDIC reduction with IMPT.

## Introduction

In patients with limited-stage Hodgkin lymphoma (HL), consolidation radiation therapy after frontline chemotherapy reduces the risk of relapse.^1^ Lymphocyte depletion has been associated with adverse clinical outcomes in HL patients,^2^, ^3^, ^4^ and the promising results of immune checkpoint inhibition in first-line systemic therapy in HL patients^5^ highlight the importance of preserving a functional immune system in HL patients. In this context, any perturbation that could impair immune activation should, in theory, be managed. Radiation therapy exposes organs at risk, and the immune system has been recognized as such, particularly in esophageal and lung cancers,^6^, ^7^, ^8^ where a higher effective dose to circulating immune cells has been shown to negatively correlate with overall survival. Recently, in patients with mediastinal Hodgkin lymphoma (mHL), proton therapy has been shown to be beneficial in terms of cardiac sparing.^9^ However, this cardiac benefit has not been systematic and has mostly been restricted to patients with lower mediastinal involvement.^10^, ^11^ However, proton therapy interest in reducing the burden on the immune system has never been evaluated in mHL patients. Therefore, this study was designed to evaluate the potential benefit of proton therapy on immune system exposure in a setting of consolidation radiation therapy.

## Material and method

This was a single institution, retrospective dosimetric study. Ten consecutive patients with unfavorable limited-stage mHL who were treated with intensity-modulated proton therapy (IMPT) in a consolidation setting after frontline chemotherapy in 2022 were selected for this study. Fully anonymized patient data were used in this study, which was reviewed and approved by the institutional review board of the Institut Curie (Paris, France).

Patients were placed in the supine position; simulation computed tomography scans were acquired with spirometer-guided deep inspiration-breath hold using 2 mm slices, and images were transferred to the Aria treatment planning system (Varian Medical System) for IMPT planning. Clinical target volumes (CTVs) were defined in accordance with the involved-site radiation therapy standard.^12^ The IMPT plan calculations used multifield robust optimization on the CTV, with an isotropic setup error of 3 mm and a range uncertainty of 3%. One to 4 pencil beam scanning fields were planned. Patients were treated to a total dose of 30 Gy in 15 fractions according to European Society for Medical Oncology guidelines using proton beams generated by a 230 MeV C230 cyclotron (IBA). Dose constraints to organs at risk were in accordance with ILROG guidelines,^13^ including mean heart dose ≤5 Gy, mean breast dose ≤4 Gy, lung V5Gy ≤55%, lung V20Gy ≤30%, mean lung dose ≤10 Gy, and thyroid V25Gy ≤62.5%. As part of the standard institutional procedure, all patients were also planned with an equivalent volumetric modulated arc therapy (VMAT) photon plan (in case of IMPT treatment room unavailability), aiming that a minimum of 95% of the prescribed dose was planned to be delivered to 95% of the planned target volume (PTV) and a maximum of 107% of the prescribed dose could be delivered to 2% of the PTV. VMAT PTV was defined with an isotropic margin of 5 mm around CTV. Dose constraints to organ-at-risk for the VMAT plans were identical to those used for proton therapy planning, with identical weighting and prioritization. In addition, the VMAT plans, although prescribed on PTV, were designed to provide the same CTV coverage as the proton therapy robust plans. The arcs used 6 MeV photons on TrueBeam linear accelerators (Varian), with a maximum dose rate set at 600 monitor unit/min and control points spaced every 4°.

**Figure 1:**
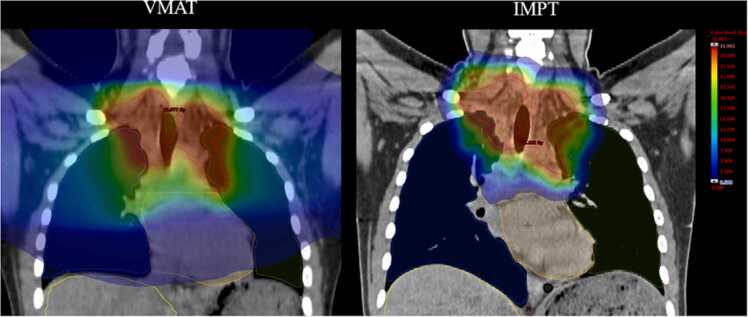
Radiation dose exposure to different organs at risk involved in effective dose to circulating immune cells (liver in yellow, heart in orange, lungs in blue and green) in a patient with mediastinal Hodgkin lymphoma treated with locoregional VMAT and IMPT. Abbreviations: IMPT, intensity-modulated proton therapy; VMAT, volumetric modulated arc therapy.

Mean doses to the heart (MHD), lungs (MLD), and liver (MlD), as well as mean dose to the partial body (partial mean total body dose [MBD]) and partial body volume (partial body volume), were obtained from dose-volume histograms in the VMAT and IMPT plans (Figure 1). The MBD was then calculated from the weight of each patient. The mean body density was set at 1.05 for a 50-year-old woman.^14^

$$\;\mathrm{MBD}=\;\frac{\mathrm{partial MBD}\times \mathrm{partial body volume}}{\mathrm{Weight}}\times 1.05.$$

All patients irradiated in a consolidation setting for mediastinal Hodgkin disease were scanned from the inferior edge of the transverse process of C2 to the inferior edge of the liver, the latter being systematically scanned in its entirety, enabling us to report the mean hepatic dose. As the anatomical limits of the simulation computed tomography scan were consequently well away from the CTV, we assumed that the dose delivered outside the scanned volume was 0.

Based on the combination of these dosimetric parameters and the number of fractions (*n*), the effective dose to circulating immune cells (EDIC) was then calculated for each patient as follows^6^:

$$\mathrm{EDIC}=0.12\times \mathrm{MLD}+0.08\times \mathrm{MHD}+0.15\times 0.85\times \sqrt{\frac{n}{45}}\times \mathrm{MlD}\;+\left(0.45+0.35\times 0.85\times \sqrt{\frac{n}{45}}\right)\times \mathrm{MBD}$$

For each patient, the relative contribution of the lung, heart, liver, and total integral dose to the EDIC was assessed, as was their respective contribution to the variation in the EDIC between IMPT and VMAT plans. A nonparametric paired Wilcoxon Mann-Whitney test was used to compare the calculated EDIC for each patient between VMAT and IMPT plans. The level of statistical significance was set at 0.05. R software (version 4.1.5) was used for all analyses.

## Results

The 10 mHL patients included in the study were all female, with a median age of 27 years (range: 22-37 years). All of them were treated with a total dose of 30 Gy (relative biological effectiveness) in 15 fractions.

**Figure 2:**
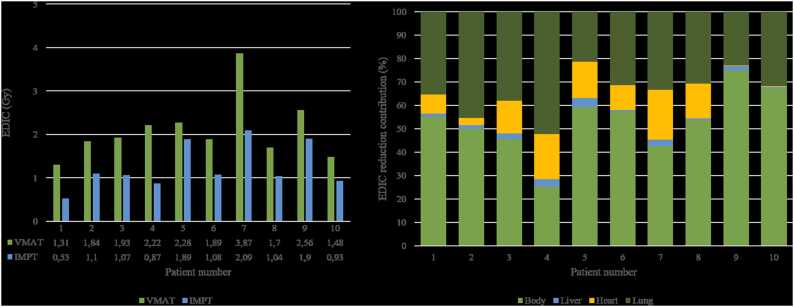
(A) EDIC (Gy) with VMAT (green) and IMPT (blue). (B) Relative contribution in EDIC reduction with IMPT of radiation sparing of the lungs (dark green), of the heart (yellow), of the liver (blue), and of the body (integral dose, light green). Abbreviations: EDIC, effective dose to circulating immune cells; IMPT, intensity-modulated proton therapy; and VMAT, volumetric modulated arc therapy.

In all patients, the EDIC was always lower with IMPT than with VMAT. The median EDIC was decreased from 1.93 Gy (range: 1.31-3.87) with VMAT to 1.08 Gy (0.53-2.09) with IMPT (*P* < .01) (Figure 2A). The median EDIC absolute reduction was −0.76 Gy (range: −1.79; −0.39), and the median EDIC relative reduction was −41.7% (range: −60.9%; −17.0%). Among the 10 patients included, all had upper mediastinal involvement, and 5 additionally had lower mediastinal disease, which was defined by a CTV that extended below the origin of the common trunk of the left coronary. The median CTV was 165.3 cm^3^ (range: 67.7 cm^3^; 295.4 cm^3^). No correlation was found between the CTV volume and either absolute or relative EDIC reduction (*P* = .103 and *P* = .728, respectively); nor was there any correlation between lower mediastinal involvment and either absolute or relative EDIC reduction (*P* = .827 and *P* = .077, respectively).

Integral dose reduction was the main driver of EDIC reduction with IMPT (Figure 2B), contributing 53.7% (25.36%-75.01%) of the gain in EDIC reduction. Lung sparing was the second most important contributor to EDIC reduction. It accounted for 33.4% (21.2%-52.1%) of the EDIC reduction.

## Discussion

The rationale for sparing the immune system during radiation therapy in Hodgkin disease derives from what has been reported in solid tumors. Cytotoxic T lymphocytes are highly sensitive cells to radiation,^15^ and lymphopenia has been associated with inferior overall survival in multiple solid tumors such as lung and esophageal cancers.^16^, ^17^ Indeed, lymphocytes play a predominant role in the antitumor response in both solid^18^ and hematological tumors.^19^, ^20^, ^21^ Consequently, exposure of effector immune cells to ionizing radiation during radiation therapy is likely to reduce the ability of the immune system to suppress tumor cells in Hodgkin disease.

Our results show that proton therapy significantly reduces EDIC in the irradiation of mHL, for which, at the moment, the majority of studies have evaluated the benefit of proton therapy in terms of reduction of cardiac exposure.^9^, ^11^, ^22^, ^23^, ^24^ Proton therapy has been associated with a lower EDIC in non–small cell lung cancer,^8^ where the detrimental effect of EDIC on survival has been well documented. Since proton therapy offers a significant dosimetric benefit in terms of immune system sparing, assuming that this immune system sparing translates into a clinical benefit, proton therapy could also improve the survival of patients treated for mediastinal Hodgkin disease, other than through a reduced risk of radiation-induced cardiac toxicity or second cancers, which have until now been the main theoretical benefits associated with proton therapy in this indication.^9^, ^25^, ^26^ It should also be pointed out that cost-economy studies have shown that younger patients require greater cardiac sparing for proton therapy in this population to be cost-effective.^24^ The possibility of better tumor control via greater sparing of the immune system may call for a potential re-evaluation of these studies, particularly for younger patients. In this case, the benefit of immune system preservation could potentially be added to the benefit of cardiac preservation with proton therapy. However, EDIC has not yet been shown to correlate with survival in HL. The existence of such an association will be evaluated with the development of comprehensive databases, including HL patients receiving immunotherapy.

The main advantage of proton therapy on EDIC is related to the reduced integral and pulmonary dose, while reducing the dose to the heart makes a similarly small contribution to reducing proton EDIC. On the other side, liver exposure has a negligible contribution in EDIC in mHL patients. Similarly, deep inspiration-breath hold treatment, usually recommended by treatment guidelines,^27^ may offer immunostimulatory benefits by reducing EDIC through mean lung dose reduction. Although no variables were found to identify a group of patients in whom proton therapy would be most beneficial in reducing EDIC, this may have been related to the small size of the study population. It is nevertheless theoretically conceivable that cardiac, pulmonary, and hepatic exposure would be most significant in patients with extensive lower mediastinal involvement and in those with the largest burden of disease.

The optimization of treatment plans on the basis of EDIC, in addition to cardiac sparing considerations, and the selection of patients for proton therapy by means of comparative dosimetry focused on this parameter are potential areas of research that could benefit from the contribution of artificial intelligence.^11^, ^28^ There are several models for the assessment of the radiation doses received by circulating immune cells during a specific RT treatment, but they are still incomplete. In particular, since the effects of the different dose rates between the different techniques have not yet been taken into account, 4D computational models could modify the results that we have obtained.^29^

## Conclusion

The dose received by the immune system during consolidation of involved-site radiation therapy for HL is statistically lower with proton therapy than with VMAT. The main reason for the reduction in EDIC with IMPT was the overall dose reduction associated with improved lung sparing.

## Funding

The authors declare that they have no funding and no financial support.

## Author Contributions

Pierre Loap: Conceptualization, Data curation, Formal analysis, Funding acquisition, Investigation, Methodology, Software, Supervision, Validation, Visualization, Writing- Original draft, Writing- Review and Editing. Ludovic De Marzi, Justine Decroocq, Rudy Birsen, Natacha Johnson, Didier Bouscary, Benedicte Deau Fischer, and Youlia Kirova: Validation, Writing- Review and Editing.

## Declaration of Conflicts of Interest

The authors declare that they have no competing interests.

## Data Availability

Research data are stored in an institutional repository and will be shared upon request to the corresponding author.
